# The quest for harmonisation in anti-doping: an Indian perspective

**DOI:** 10.1007/s40318-022-00220-7

**Published:** 2022-05-25

**Authors:** Shaun Star

**Affiliations:** 1 Professor, Jindal Global Law School, O.P. Jindal Global University, Sonipat, Haryana India; 2 Ph.D. Candidate, TC Beirne School of Law, The University of Queensland, St Lucia, QL Australia

**Keywords:** Anti-doping, Court of Arbitration for Sport (CAS), National Anti-Doping Organisations (NADOs), World Anti-Doping Agency (WADA), India, Harmonisation

## Abstract

The World Anti-Doping Agency aims to promote clean sport through the introduction and implementation of harmonised rules under the World Anti-Doping Code, 2021 (the Code). Since WADA relies heavily on National Anti-Doping Organisations to implement the Code, the experience of anti-doping differs across countries. Some scholars argue that the current framework disproportionately impacts athletes from developing countries. This paper contributes to this debate by analysing systemic issues in the implementation of the Code in one such country—India. The legitimacy of anti-doping in India has been questioned as a result of the recent suspension of the National Dope-Testing Laboratory, a series of false positive tests, accusations of significant procedural and substantive errors by domestic tribunals, and access to justice challenges. Given the prevalence of doping in India, alongside the accumulation of recent controversies and push for reform, a deeper analysis of anti-doping in the country is warranted. The lack of compliance in India with certain requirements set out in the Code, as well as the failure to meet “best practice” standards set by other jurisdictions, is evidence that there is a lack of harmonisation in implementing anti-doping rules and procedures across countries. This paper contributes to the debate on the impact that a lack of harmonisation in the implementation of the Code can have on the legitimacy of the anti-doping framework. From a policy perspective, the proposed research agenda and recommendations can be applied to promote reform in India and other jurisdictions, especially in developing and emerging countries.

## Introduction

Doping threatens to undermine the spirit of fairness that underpins sport. Accordingly, governments and international organisations have built a framework to restrict, deter and sanction doping in sport. The World Anti-Doping Agency (WADA) aims to promote clean sport through the introduction and implementation of harmonised rules under the World Anti-Doping Code, 2021 (the Code).^1^ The implementation of the Code relies on the cooperation of national governments which ratify the provisions of the Code according to their specific constitutional requirements.^2^ WADA has acknowledged that “a central pillar” of its mission is to monitor signatories to ensure that they are in compliance with the Code so that “all countries follow the same set of rules and implement compliant anti-doping programs”.^3^ According to WADA, uniform compliance by all signatories is critical for the anti-doping system since “harmonization means that athletes know what to expect from the anti-doping system no matter where they are from or where they are competing.”^4^ While the Code has now been adopted by most international sport federations and national governments, the experience of anti-doping differs across countries.^5^ To date, most scholarship on the effectiveness of the implementation of anti-doping policy has focussed on developed countries such as the UK.^6^ However, scholars have argued that the current framework disproportionately impacts athletes from developing countries^7^ and, therefore, any holistic discussion on the effectiveness of the harmonisation of the anti-doping system ought to take into account the implementation of the Code in such nations. Limited research has been conducted on the impact of the adoption of the Code in developing and emerging countries.^8^ This paper contributes to this debate by analysing systemic issues in the implementation of the Code in one such country—India. Indeed, India has a high prevalence of anti-doping rule violations amongst its athletes, with the majority of athletes’ cases being determined by domestic tribunals.^9^ Recently, the legitimacy of the anti-doping framework has been questioned as a result of the suspension of the National Dope-Testing Laboratory (NDTL),^10^ a series of highly publicised false positive tests,^11^ accusations of significant procedural and substantive errors by domestic tribunals,^12^ and access to justice challenges before the High Court.^13^ In addition, there has been discussion of anti-doping reform, with the introduction of the National Anti-Doping Bill (2021), in Indian Parliament, as well as separate government policy with respect to regulation of supplements^14^ which have been the cause of many inadvertent anti-doping rule violations in India. Given the prevalence of doping in India, alongside the accumulation of recent controversies and push for reform, a deeper analysis of anti-doping in the country is warranted. The paper will first set out the anti-doping framework and discuss the importance of the quest for harmonisation in promoting legitimacy of anti-doping institutions. Second, the paper provides an overview of anti-doping in India and discusses the current systemic challenges with respect to the implementation of anti-doping policies within the country. Following this, the paper adopts a case study approach to highlight the practical application of these challenges. Finally, the author sets out recommendations and areas for potential reform to anti-doping in India. It is argued that the lack of compliance in India with certain requirements set out in the Code, as well as the failure to meet “best practice” standards set by other jurisdictions, is evidence that there is a lack of harmonisation in implementing anti-doping rules and procedures across countries. This paper contributes to the debate on the impact that a lack of compliance and harmonisation in the implementation of the Code can have on the legitimacy of the anti-doping framework. From a policy perspective, the discussions, proposed research agenda and recommendations can be applied to promote positive reform in India and other jurisdictions, especially in developing and emerging countries.

## The anti-doping agenda

### The regulatory framework

The purpose of the Code is to ensure universal harmonisation of anti-doping with respect to detection, deterrence and prevention of doping.^15^ The Code sets out specific anti-doping rules that National Anti-Doping Organisations (NADOs) are responsible for adopting, implementing and enforcing within their authority.^16^ While the Code allows national agencies some flexibility in the rules that they adopt in their respective jurisdictions, there are a number of articles of the Code which are mandatory and must be adopted by each anti-doping organisation without any substantive changes.^17^ While each jurisdiction may establish its own dispute resolution infrastructure to hear disputes with respect to anti-doping rule violations (ADRVs), these matters may ultimately be appealed to the Court of Arbitration for Sport (CAS).^18^

NADOs play a crucial role in implementing the Code and ensuring compliance with the anti-doping rules across different countries. In India, the National Anti-Doping Agency (NADA) governs anti-doping in sports. The National Anti-Doping Rules, 2021 (NADA Rules) set out the procedure for the collection of samples, the management of test results, and the conduct of hearings at the national level. The rules are similar to the provisions of the Code. On paper, anti-doping institutions and procedures in India are similar to most jurisdictions around the world. However, due to the flexibility and autonomy afforded to NADOs, there are differences in the implementation of different aspects of the Code, including with respect to testing, education, and some procedural elements.

### The quest for harmonisation

WADA’s primary goal is to promote a harmonised anti-doping system. For the anti-doping framework to create a level playing field, it is important that NADOs implement the Code consistently. This is especially true with respect to testing procedures, upholding the rights of athletes, and handing down proportionate sanctions in accordance with the Code. However, the implementation of the Code varies from jurisdiction to jurisdiction and significant variations even exist among NADOs that are considered ‘global frontrunners in the struggle against doping’.^19^ Gray has argued that the three factors that have hindered compliance with the Code, and therefore inhibited harmonisation of anti-doping policy, are the top-down approach to implementation, cultural variations, and lack of resources.^20^ First, with respect to WADA’s top-down approach, Gray notes that WADA’s sanctions are limited to withdrawing laboratory accreditations, and it relies on other stakeholders, including the International Olympic Committee, international federations and NADOs, to ensure compliance with the Code.^21^ Second, cultural variations present a challenge to compliance since ‘[d]ifferent geographical and cultural contexts affect the way in which international agreements are absorbed and interpreted’.^22^ Many have argued that anti-doping regulations are designed, interpreted, and enforced by those in the Western culture,^23^ and that to ensure compliance and harmonisation such policies need to be effectively ‘translated and embedded into non-Western cultures’.^24^ This is a significant challenge for WADA. Third, when translating these rules and procedures into different geographical and economic contexts, it needs to be acknowledged that some NADOs are better resourced than others. In some countries—especially developing and emerging nations—implementation of anti-doping policy is not high on the nation’s policy agenda. Consequently, resource limitations in certain parts of the world, including in parts of South Asia, Africa, South America and Eastern and Central Europe, NADOs simply ‘do not have the capacity to comply’.^25^ In fact, the Chief Executive Officer of the Anti-Doping Authority Netherlands noted that ‘the Code demands much more than even the most developed NADOs can realise’.^26^ Gray’s three factors provide a useful framework on which to analyse compliance in anti-doping, especially with respect to testing standards, procedural fairness and education. Compliance with strict testing standards relies heavily on what Gray defines as WADA’s top-down approach, yet as discussed throughout this paper due to cultural differences and resource constraints, testing standards may vary (albeit in exceptional circumstances) across jurisdictions. Similarly, given the reliance on NADOs (and tribunals) to provide procedural protections to athletes, WADA’s top-down approach has some limitations with respect to ensuring procedural compliance by national tribunals, as well of education of athletes. Cultural variations and resource constraints play a significant role in the lack of uniformity with respect to procedural fairness and education of athletes. It is clear that despite WADA promoting the goal of harmonisation that the consistent and harmonised implementation of the Code is a significant challenge for the anti-doping ecosystem.

Compliance with the Code by all stakeholders is critical in the quest for harmonisation in anti-doping policy. The extent to which WADA and NADOs successfully implement the Code ‘is a determining feature of [their] legitimacy and capacity to accrue support from [their] various audiences’.^27^ Pielke Jr. and Boye argue that scientific integrity should be a guiding light in the implementation of the Code and that it ‘underpin[s] the legitimacy of anti-doping regulation.’^28^ Without scientific integrity, the quest for harmonisation will surely fail. For instance, accredited laboratories must apply the same high standards for testing, irrespective of where they are based, to avoid inaccurate or inconsistent results. Anti-doping panels need to comply with strict substantive and procedural compliance requirements to ensure due process, ensuring that the rights of all athletes are upheld. However, scholars have argued that case studies suggest that stakeholders have ‘… departed significantly from a grounding in scientific evidence’ and that this is ‘… reflective of systemic shortfalls in anti-doping regulation’.^29^ Keeping in mind the importance of consistency and scientific integrity in achieving the quest for harmonisation, it is an important exercise to critically examine the extent to which different stakeholders comply with the Code. As discussed above, while scholars and practitioners have claimed that harmonisation has not been achieved across different countries, there is scope for further analysis on the extent of compliance with the Code by specific countries. Given the strong arguments that the Code effects NADOs and athletes from developing countries disproportionately, a case study analysis of compliance with the Code in a country such as India may shed some light on how far the quest for harmonisation is from being realised.

## Anti-doping in India: context and current challenges

### Anti-doping rule violations

**Figure 1:**
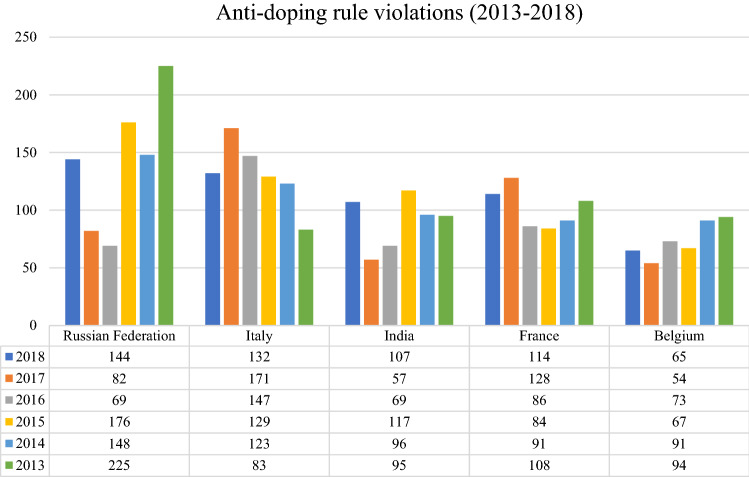
Total ADRVs from 2013 to 2018 in Russia, Italy, India, France and Belgium. The data with respect to the total number of ADRVs in each country has been compiled based on the annual ADRV reports released by WADA. See, WADA (2015); WADA (2016b); WADA (2017); WADA (2018); WADA (2019a); WADA (2020a)

Doping is prevalent in Indian sport. Since 2009, NADA has tested more than 40,000 athletes for ADRVs and a total of 1206 athletes have committed anti-doping rule violations under the NADA Rules.^30^ India has consistently ranked as one of the worst offenders in the Anti-Doping Rule Violation (ADRV) reports published by WADA.^31^ In 2018, Russia (144 ADRVs), Italy (132 ADRVs) and France (114 ADRVs) topped the list of doping violations, and India (107 ADRVs) was a close fourth.^32^ In 2017, India had the fourth highest number of ADRVs (57)^33^ and for the 3 years prior to this, India had the third highest number of ADRVs for 3 years in a row (2015-2017).^34^ Figure 1 shows the number of ADRVs in India over the past 5 years relative to other countries which consistently rank high on WADA’s ADRV list. A perusal of the ADRV list published on the NADA website shows that more than 98% of ADRVs are a result of an athlete testing positive for a prohibited substance, and therefore in violation of Article 2.1 of the Code.^35^ Several athletes were in violation of the NADA Rules due to their refusal or failure to provide sample. While limited empirical evidence exists with respect to the prevalence of inadvertent doping in India, anecdotal evidence and commentary suggests that it is widespread with lack of awareness, or inadvertent consumption of a banned substance due to medicine or supplement usage being common.^36^ On rare occasions where an athlete’s sample has been retested by a foreign lab and found to have been a “false positive”,^37^ such athletes appear to have been removed from NADA’s ADRV list despite (wrongfully) having served a suspension.

**Table 1:** Number of tests administered by NADOs and percentage of adverse analytical findings (AAFs) (2015–2020) (*n* = total tests)

NADO	Year											
	2020		2019		2018		2017		2016		2015	
	*n*	% AAFs (%)	*n*	% AAFs (%)	*n*	% AAFs (%)	*n*	% AAFs (%)	*n*	% AAFs (%)	*n*	% AAFs (%)
India	1186	4.6	4004	5.6	3979	2	3174	2.2	2831	2.6	5162	2.1
Iran	370	4.1	1214	5.3	795	4.5	1149	4.6	997	4	888	4.8
South Africa	358	3.1	1542	3.8	1124	3.1	1178	3.8	2795	2.6	2667	2.7
USA	7756	1.8	11213	1.7	9958	1.5	9820	1.4	9131	1.5	7547	1
Belgium (Flanders)	1345	1.7	1933	1.7	1914	1.8	2059	1.9	1919	2.4	2082	2.8
Canada	1225	0.5	3902	1.1	3404	1.5	3943	1.4	3443	1	2797	1.3
Italy	5043	0.4	8539	1	8587	1	8710	0.9	8158	1	5377	0.6
Russia	6861	0.8	9516	0.9	8322	1.4	5487	0.6	2557	2.6	12536	1.2
France	6104	0.5	7388	0.9	7669	1.7	7276	2.3	7457	2.1	7141	1.7
Australia	2685	0.6	4729	0.7	4542	0.6	4641	0.9	4430	1.2	4631	0.5
New Zealand	1012	0.4	1302	0.5	1272	0.6	1688	0.5	1232	0.6	1087	0.6

In addition to the high prevalence of doping in India, and the need for better education, the anti-doping system has been rife with controversy in India in recent years. The following sections discuss issues with respect to testing, procedural and substantive errors in panel decisions, and concerns with respect to inadvertent doping and anti-doping education. Despite efforts to promote harmonisation in anti-doping procedures globally, shortcomings in testing, procedural fairness and education each threaten to undermine the legitimacy of anti-doping in India.

### Testing

#### Testing trends in India

In India, NADA is responsible for the testing of its athletes, whereas the National Dope Testing Laboratory (NDTL) in New Delhi conducts analytical testing of samples to determine whether they contain any prohibited substances. All accredited testing laboratories are required to ensure that they comply with the provisions of the International Standard for Laboratories (ISL), in particular those requirements set out in Article 4.4.^38^ Compliance with these rules is mandatory so as to ensure that these laboratories consistently produce valid test results, and as these rules promote a testing system that is uniform and harmonised, regardless of where a dope test takes place.^39^ In August 2019, NDTL’s WADA accreditation was suspended due to non-compliance with the ISL and its corresponding technical documents.^40^

As set out in Table 1, testing numbers are not high compared to other jurisdictions, especially given India’s large population. The number of athletes that NADA has tested decreased significantly from 2015 (5162 samples) to 2016 (2831 samples), despite 2016 being an Olympic year. The number of athletes tested has slowly increased to 4,004 samples in 2019. As was evident across most jurisdictions, the impact of the COVID-19 pandemic resulted in a significant decrease in testing in 2020, with a total of 1186 tests being conducted throughout the year. This is perhaps expected given that (1) India was in a nation-wide lockdown for much of 2020, (2) the NDTL was suspended for this period, requiring all dope tests to be sent abroad for testing at a considerable expense for NADA. As a result of the relatively low testing levels, the percentage of athletes returning a positive adverse analytical finding (AAF) is much higher than other countries, and indeed it has increased significantly recently. In 2019, the percentage of athletes returning a positive AAF was 5.6%, and in 2020, 4.6%, both well above the international average.

This trend of decreasing tests and an increased percentage of AAFs, was opposite to the global trend prior to the COVID-19 pandemic. Globally, there was a slight decrease in the total percentage of AAFs, from 1.05% in 2018 (2774 AAFs from 263,519 samples) to 0.97% in 2019 (2702 AAFs from 278,047 samples).^41^ Conversely, in India, the percentage of AAFs more than doubled from 2018 to 2019, increasing from 2% to 5.6%. In 2020, there was a 46.1% decrease in the number of samples analysed globally as a result of the COVID-19 pandemic, and a decrease in the total percentage of AAFs 0.67% (1009 AAFs from 149,758 samples),^42^ whereas India’s testing decreased by more than 70% and the percentage of AAFs decreased by 1%. Even prior to the impact of the pandemic, the number of tests in India had decreased and despite this the percentage of AAFs was increasing. While the reduced number of tests in India, and consequent increase in percentage of AAFs, might on the face of it show a decline in the number of athletes testing positive to a banned substance, it may also indicate that there are likely more athletes who are not being caught. While the decrease in testing during the pandemic was consistent with global trends, there is no evidence available as to why testing decreased so significantly from 2015 onward and only gradually increased in subsequent years. In response to criticisms that NADA reduced testing drastically from 2015 onward, a NADA official argued that “it is not the quantity of the tests that is material” but rather the quality, further explaining that “[i]n 2015 it was the run-up to the Olympics, so a large number of tests had to be conducted.”^43^ However, with the exception of Russia, no other jurisdiction experienced such a significant decline in testing after 2015. In fact, the data in Table 1 indicates that many countries increased testing from 2015 to 2016,^44^ while according to WADA, there was an overall decrease in the number of samples from 2015 to 2016 of only 0.9% (303,369 in 2015 to 300,565 in 2016).^45^

It should be noted that while most developed countries consistently returned a percentage of AAFs of lower than 2% in recent years (see Table 1), some developing countries also showed higher percentages similar to India (for instance, the average percentage of AAFs in Iran was 5.82% from 2015 to 2020, while South Africa averaged 3.13% during the same period). While neither Iran nor South Africa returned the large number of ADRVs as has been the case in India in recent years, the fact that South Africa, Iran and India all have a higher percentage of AAFs than the developed counties listed in Table 1 perhaps points to the need for further research (with a larger sample size) to understand whether there are consistent trends when comparing developed and developing countries with respect to the percentage of AAFs.

#### Consequences of the suspension of NDTL

WADA can suspend or revoke the accreditation of a lab if it fails to comply with the ISL or technical documents.^46^

In September 2018, WADA listed several major objections with NDTL, including issues with its isotope ratio mass spectrometry (IRMS) sampling procedure,^47^ faulty standard operating procedures with respect to the testing, and an inefficient quality management team.^48^ Based on the recommendation of an independent disciplinary committee, WADA suspended NDTL’s accreditation in August 2019 for non-compliance with the ISL.^49^

Commentators were not surprised with NDTL’s suspension given that ‘WADA had been giving repeated warnings to the NDTL to bring its testing methods in line with ISL and the related Technical Documents’.^50^ For instance, six tests which returned a negative AAF were retested in the WADA-accredited Montreal lab in Canada which found them to be positive,^51^ resulting in those six Indian athletes being suspended. NDTL also returned several false positives, whereby athletes were wrongfully suspended based on erroneous results from the NDTL. When the samples of four athletes were retested by the WADA-accredited laboratory in Rome, they were found to be negative.^52^ In 2016, the initial negative report of an Indian athlete was overturned by the Cologne laboratory on conducting an IRMS analysis.^53^ The consequences of errors in the results management process can be significant for an athlete.

Athletes have also claimed that there have been procedural shortcomings in the result management process, including issues with respect to the chain of custody after samples have been collected. For example, in Inderjeet Singh’s case it was alleged (and originally conceded by the doping control officer) that the accused athlete’s urine sample had been stored in the doping control officer’s home refrigerator overnight.^54^ In 2021, the ADAP cleared Vijay Singh, an amateur Indian athlete, of his suspension after NADA was ordered to retake his urine sample and send it to the WADA-accredited laboratory in London.^55^ The athlete alleged that several unauthorised people were present at the Doping Control Station throughout the sample collection process which was in violation of the Urine Sample Collection Guidelines.^56^ The Delhi High Court also emphasised the importance of avoiding delay in the results management process, noting that:

*Testing of samples in a timely manner is crucial as sportspersons like the [athlete] are placed on a ban in the interregnum and the … National Anti-Doping Agency ought to act with urgency while dealing with such matters.*^57^

Following the high court order, and after comparing the DNA in each of the samples, the report from the laboratory showed that the original urine sample on which the athlete’s AAF was based was not in fact the athlete’s urine.^58^ Despite being ineligible to participate in sport for approximately 2.5 years, the ADAP held that the athlete had been wrongfully sanctioned based on a positive AAF from another athlete’s sample.^59^ Highlighting the importance of the results management procedure, the ADAP also acknowledged that NADA’s doping control officers need to strictly comply with the standard operating procedures of sample collection.^60^

NDTL’s suspension has added to the difficulty of the results management process during the COVID-19 pandemic. There has been a significant increase in the cost of transporting the samples to international laboratories^61^ which is likely to result in a lower number of tests being conducted given the NADA’s limited budget.^62^ It should be acknowledged that in December 2021, WADA restored the accreditation of NDTL noting that it had now become fully compliant with the ISL.^63^

### Procedural issues in anti-doping disputes

In India, the Anti-Doping Disciplinary Panel (ADDP) has been constituted to hear disputes with respect to ADRVs by athletes.^64^ Cases may be appealed to the Anti-Doping Appeal Panel (ADAP) and cases arising from international events or involving international athletes may be appealed to the CAS, after an appeal to ADAP.^65^ An international level athlete may also request a single hearing before CAS, with WADA and NADA’s consent, rather than exhausting the hearing process at a domestic level.^66^

Commentators have argued that systemic issues exist in some first-instance tribunals when an athlete’s alleged ADRV is being heard.^67^ This is particularly true of hearings in India where there have been allegations of substantive errors made by the ADDP, access to justice complaints, and significant delays in hearings, all of which are critical given how much is at stake for athletes accused of an ADRV.

The ADDP has been accused of erroneous decisions. For instance, Rajaraman argues that the ADDP ‘mixed up cases’ as evidenced by the fact that ‘a panel copied–pasted a paragraph from an earlier order’, despite such circumstances not being applicable to the case.^68^ Mohan alleged that the ADDP and ADAP have regularly applied 4-year sanctions to athletes who have tested positive to a specified substance out of competition (an ADRV that typically results in a maximum ineligibility period of 2 years, unless NADA can prove that the athlete intentionally consumed the banned substance for performance enhancing purposes).^69^ It appears that the panels accepted NADA’s arguments that not listing the supplements or medicines on the athlete’s doping control form was sufficient evidence of the athlete’s intention to cheat.^70^ Commentators argue that there have been ‘glaring disparities’ in the way panels interpret the rules, both within India and compared to other jurisdictions, and that consequently WADA should ‘hold workshops and seminars for the benefit of those who determine the fate of athletes’.^71^

Minimum procedural guarantees exist under the Code, including an athlete’s right to a fair, impartial and independent hearing, the right to legal representation, the right to an accessible and affordable hearing process, and the timely resolution of disputes.^72^ However, it has been argued that first-instance hearings in developing countries might fall short of these procedural guarantees more often than their counterparts in developed countries.^73^ This is evidenced by the fact that countries such as Australia, New Zealand, and the UK have constantly implemented reforms with respect to their sports dispute resolution procedures, whereas countries such as India have not.^74^

Although athletes have the right to be represented by counsel at their own expense,^75^ access to justice issues exist in anti-doping disputes for many athletes around the world given the affordability of counsel, expert evidence and laboratory analysis.^76^ However, such access to justice issues are more pronounced in developing countries.^77^ A perusal of the publicly available decisions handed down by the ADDP shows that many athletes are unrepresented at first instance. This, coupled with the fact that commentators have argued that there have been systemic issues of delay and access to justice at first-instance hearings in India,^78^ are problematic. While many athletes have the right to appeal to the CAS, this right is rarely exercised by Indian athletes. In fact, only 14 athletes (of the 1206 ADRVs in India) have had their cases heard by the CAS. All but one of these cases were appealed by WADA to the CAS. It has been previously argued that ‘the fact that only one Indian athlete has ever appealed their case to the CAS may in itself be prima face evidence of access to justice issues in the anti-doping dispute resolution framework’.^79^

Athletes are entitled to ‘a fair hearing within a reasonable time’^80^ and under the International Standard of Results Management (ISRM) strict timelines must be followed by first-instance tribunals.^81^ Under all previous versions of the NADA Rules, strict time limits were also provided. For instance, under NADA Rules, 2015, Article 8 prescribed a 45-day time limit between the constitution of the panel and the hearing, and a 90-day time limit between the constitution of the panel and the written decisions. However, such time limits are often not followed, and athletes have regularly complained of significant delay in the results management process, including the hearing process, in India.^82^ For instance, in *NADA v Anil Kumar* (2012), more than 1000 days passed between the athlete being tested to a first-instance decision of the ADDP.^83^ In this case, the athlete’s sample was collected at a selection trial for the World Cup Kabaddi 2010 on 20 March 2010, where a banned substance was found in his system, and the first-instance panel ultimately made a decision on 27 December 2012.^84^ Curiously, the athlete was notified of the result of their B sample analysis on 14 May 2010 and was only notified of the constitution of the panel on 30 November 2012 (two and a half years later). There is no justification given for this significant delay in the decision of the ADDP.

To properly assess whether any systemic issues exist in terms of the timeliness of the results management system in India, a thorough empirical study should be completed. What is clear, however, is that these extended (unexplained) delays are not acceptable, both under the Code and NADA Rules.

### Education and inadvertent doping

Education is a central focus to WADA’s anti-doping strategy.^85^ It has been argued that the two most significant reasons for the high incidence of doping amongst Indian athletes are lack of education, and inadvertent doping due to contamination.^86^ Athletes have a duty to make sure any prohibited substances do not enter their system, and if they ultimately test positive for a prohibited substance, they will be strictly liable for an ADRV.^87^ However, there have been numerous cases where athletes have ‘been advised by doctors or pharmacists to take a particular medicine for genuine ailment’.^88^ In addition, Indian athletes place a strong reliance on the advice of coaching staff and support personnel.^89^ The risk of inadvertent doping is arguably higher in developing countries where lower literacy levels and standards of anti-doping education exist, as well as cultural issues where a strong reliance on medical professionals and coaches prevails. In any event, studies have shown that “[t]he absence of knowledge about the possible dangers of nutritional supplements might lead to unintentional doping cases.”^90^ As such, it has been suggested that education of athletes and support personnel need to be scaled up in India, including at grass roots levels where doping in sport is allegedly a serious problem.^91^

Since 2016, NADA has worked closely with the Sports Authority of India (SAI) and national sport federations to increase anti-doping education and awareness programmes. However, Anish Dayal, a senior barrister in India who has represented numerous athletes in doping disputes, notes that ‘current efforts are inadequate’ and that ‘any anti-doping initiative should aggressively focus not only on detection but also on education and awareness. Athletes, support staff, federations, sports medical personnel must be equipped with well-conceived literature, consultation and workshops’.^92^

A survey of elite athletes in India published in 2022 showed that of the 181 athletes surveyed, only 38.1% had attended anti-doping education sessions hosted by NADA or their federation in their institute or training camp. Overall, 67.4% of the athletes were aware about NADA or WADA, and 53.6% were aware of suspensions for anti-doping rule violations.^93^ There was a significant increase in awareness of the risks and consequences of doping reported by those who attended these sessions, compared to those who had not.^94^ The results of this study reflect the arguments by commentators suggesting that there is significant room for improvement with respect to anti-doping education in India.^95^

### Summary

The large amount of ADRVs and a high percentage of AAFs represent a significant challenge for Indian institutions which promote clean sport. In addition, current challenges in ensuring uniformity and accuracy in anti-doping procedure are important in protecting athletes and promoting the legitimacy of anti-doping institutions in India. The following section sets out recent case studies that highlight key hurdles existing in the anti-doping framework in India. Ensuring that best practice standards in anti-doping are implemented domestically is not only in India’s interest, but the consistent implementation of the Code could also enhance the legitimacy of the global anti-doping system. Shortcomings with respect to testing, procedural irregularities and education are not uncommon in India,^96^ and a review of recent case studies highlights the implications of these inadequacies in practice.

## Case studies

To illustrate the significance of the challenges that exist in the anti-doping system in developing nations such as India, this section explores two case studies. First, the case of Dharam Raj Yadav highlights the impact of mistakes in the results management process, as well as the access to justice obstacles of athletes when faced with an ADRV. Second, the case of Amar Muralidharan explores criticisms of delay and procedural fairness in anti-doping disputes in India.

### Case 1: False positives and access to justice—Indian athletes take NADA to court

Inaccurate testing results can lead to significant consequences to athletes, especially if a false positive is returned and athletes are erroneously suspended from their sport.

A number of writ petitions have been filed in the Delhi High Court alleging that the results management process violated the applicable law and the Indian Constitution as it involves significant access to justice issues and unreasonable delay.^97^ In a writ petition filed by Dharam Raj Yadav, the athlete alleged that despite the applicable law requiring ‘to send all the relevant documentation and samples to the Sample Collection Authority “as soon as practicable” after the completion of the sample collection session’, there was a delay of more than 4 months between sample collection and the date of testing the athlete’s A sample.^98^ The athlete tested positive for a prohibited substance and the ADDP determined a period of ineligibility of 4 years. WADA ordered a re-analysis of the sample in another WADA-accredited laboratory which returned a negative result. As such, the period of ineligibility was lifted almost 1 year after the start of the suspension.

The athlete also alleged that he was denied access to justice because of the onerous provisions of the NADA Rules requiring a significant payment to be made in order to obtain NDTL’s laboratory report on the athlete’s sample. Without access to ‘the Laboratory documentation package of their sample [the athlete is] effectively denied the opportunity to adequately contest the anti-doping rule violation’.^99^ It was argued that many athletes would be unable to afford the lab report and that this requirement of payment therefore ‘militates against the principle of equality enshrined under Article 14 of the Constitution of India’.^100^ Yadav argued that the ability to obtain copies of the laboratory documentation package is a crucial part of his fundamental right to know that the laboratory has strictly observed the mandatory safeguards prescribed under the applicable rules. He argued that he was denied his right to a fair trial and not afforded due process, alleging that:

*An important facet of the right to a fair trial includes informing the accused of the accusations against him in advance. This is done by supplying him with the copies of all the evidence against him. The evidence must be supplied to him free of cost.*^101^

Yadav was suspended for 1 year, until a re-analysis of the sample showed that the testing authorities had made a mistake. He was unable to compete during this period, thus highlighting the significant impact that substantive and procedural errors can have on an athlete’s career.

Access to justice issues remain under the revised NADA Rules^102^ as an athlete is required to pay a fee for procurement of the laboratory documentation package, and an athlete typically requires an (often expensive) expert to interpret such documentation. Without access to the laboratory reports, and experts who can interpret them, an athlete places all of their trust in the legitimacy and accuracy of the system. In Dharam Raj Yadav’s case, as was the case for the other petitioners, this trust was misplaced.

Scholars have argued that under the current anti-doping system it is ‘almost impossible’ for athletes to prove that they have not committed an ADRV.^103^ This is particularly true where an athlete alleges that a false positive test has occurred since ‘evidence must be shown that the test procedure results are unreliable and that false positives occur’ and the ‘threshold for this is high’.^104^ Under the Code, the burden is on the athlete to establish, by a balance of probability, that there has been a departure from the ISL that could have reasonably caused the AAF.^105^ Given the difficulties encountered by athletes in terms of access to laboratory reports, as well as experts to interpret those reports, and legal counsel to argue them before a tribunal, this threshold would appear to be out of reach for most athletes. If we accept that false positive tests do occur, and that access to justice issues exist in certain jurisdictions, this creates a significant issue for athletes who are wrongly accused of doping.^106^ This underscores the need for compliance with respect to testing procedures, as well as due process rights, without which the quest for harmonisation falls short.

### Case 2: excessive delays and procedural fairness: Amar Muralidharan’s case

Despite express provisions in the Code and NADA Rules, NADA and the ADDP have been criticised for unjustified delays, which undermine the procedural rights of athletes.

In the only appeal by an Indian athlete to the CAS, issues of undue delay were raised by Indian swimmer, Amar Muralidharan. In this case, CAS criticised NADA for not complying with the minimum procedural guarantees under the NADA Rules.^107^ Muralidharan claimed that procedural irregularities existed in the results management process, including an alleged breach in the chain of custody and unusually long transportation time.^108^ Despite these allegations, the ADDP and ADAP both determined that the athlete should be suspended for 2 years.

The delays in the results management process are summarised in the CAS award, which notes that:

*… the provisions of Articles 8.3 and 13.6.8 of the NADA ADR (as well as Article 8.1 of the WADA Code) have not been complied with by the NADA. The Appellant was notified of the anti-doping rule violation on 20 September 2010. The Appellant was then heard for the first time two years later on 21 September 2012. Moreover, following a series of other delays in the issuance of the award following the ADDP Decision, the Appellant’s appeal was heard on 13 March 2014 – more than four months after receiving the complete ADDP Decision and more than 13 months after the required deadline under the NADA ADR. This means, the Respondents undisputedly violated the Appellant’s right to a procedure in line with the timing requirements described above*.^109^

While the arbitrator held that ‘NADA showed an alarming inability to effectively, timely, and appropriately handle the Appellant’s case’,^110^ Muralidharan’s suspension was ultimately upheld.

While this case study is perhaps an example of unusually prolonged procedural delay, it highlights the need for fast and effective decision-making procedures at a domestic level in India, which is unfortunately not always the case in anti-doping disputes. Upholding minimum procedural safeguards afforded to athletes is critical regardless of the cultural variations or resource constraints of a particular jurisdiction.

These case studies illustrate how shortcomings in a NADO’s implementation of the Code exist in an Indian context, and how they directly impact athletes. While these case studies highlight the fact shortcomings exist with respect to testing, access to justice issues and delay, empirical research is required to analyse whether these are systemic issues which disproportionately impact athletes from developing countries, including India. The following section will explore some of these issues in more detail and discusses possible areas of reform.

## Need for reform

NADA sets out its ‘primary functions’ as: (1) implementing the Code to achieve compliance by all Indian sports organisations, (2) coordinating dope testing programmes, (3) promoting research and education on anti-doping to inculcate the value of dope free sports, and (4) adopting best practice standards and quality systems to enable effective implementation and continual improvement of the anti-doping programme.^111^ NADA has fallen short in the implementation of these functions.

While similar shortcomings may exist in other developing countries, the sheer number of ADRVs in India, and the systemic challenges faced by the anti-doping institutions mean that reform is necessary and urgent. However, while WADA promotes harmonisation of anti-doping systems by NADOs, it needs to be acknowledged that implementation of a domestic anti-doping system requires considerable resources. Developing countries are likely to face resource constraints more than developed countries with respect to the anti-doping reforms required to meet best practice standards.^112^

From India’s perspective, there is significant scope for further reform in the areas of anti-doping education, mitigation of risks of inadvertent doping (especially with respect to supplement consumption), adopting best practice standards with respect to procedural fairness norms and testing procedures, as well as potential legislative and institutional reform. This section identifies areas of potential reform to India’s anti-doping system.

### Testing

From the perspective of harmonisation, compliance with testing standards is critical. Given the importance of consistency in testing across countries for the legitimacy of the system, it is not uncommon for WADA to suspend the accreditation of laboratories for non-compliance with its testing standards. For instance, in 2010 WADA suspended Malaysia’s laboratory for non-compliance with testing standards, including for false positive AAFs.^113^ NDTL’s failures in testing procedures are cause for concern for anti-doping efforts in India, and globally.

Under the current framework, the consequences for non-compliance with testing standards are a suspension or revocation of that laboratory’s accreditation.^114^ While NDTL’s suspension in India was clearly justified, it has the potential to cause broader impacts on the implementation of the Code in India. As a consequence of WADA’s suspension of NDTL’s accreditation, all samples of Indian athletes were sent to overseas testing laboratories, which was an additional cost to NADA.^115^ NADA has a limited annual budget, and as a result of the increased cost for sample analysis, there was likely to be fewer athletes tested, that too in an Olympic year.^116^ However, the cost of NDTL and NADA making mistakes is much higher. The system cannot afford false positives as this has the potential to ruin an athlete’s career, and it undermines the legitimacy and trust in the anti-doping system.

It has previously been argued that if non-compliance with the Code or testing standards is due to a lack of resources in a particular country, more focus should be on capacity building of institutions, rather than sanctions.^117^ While significant investment may be required for building capacity, there may also be other means. For instance, Müller suggests that an institutionalised mentoring programme be implemented requiring NADOs to ‘cooperate with one or two other NADOs to facilitate exchange programs and external audits … to enhance quality and harmonisation’.^118^ While NADA signed a 2-year memorandum of understanding alongside the Australian Anti-Doping Agency (ASADA) and WADA to ‘ensure India implements a more effective anti-doping program that is fully compliant with the [Code]’,^119^ there is scope for deeper collaboration with other NADOs to share knowledge and promote best practice in the results management process. In any event, it would be prudent for the Ministry to invest in NDTL to ensure that it can further upgrade its equipment as well as build capacity through investing in staffing requirements, to guarantee compliance with WADA’s testing standards.^120^ To this end, WADA needs to continue implementing its top-down approach to not only sanction laboratories who fail to meet standards, but also build capacity and accountability mechanisms to ensure continued compliance and uniform best practices in testing in all laboratories, in all circumstances.

### Compliance with procedural standards

WADA and NADOs can do more to ensure compliance with procedural guarantees in the results management process.^121^ NADA and India’s anti-doping panels have been criticised for their lack of procedural compliance previously.^122^ NADA should ensure compliance with the procedural safeguards now enshrined under the ISRM and Article 8 of the Code. In addition, there are various procedural reforms that other jurisdictions have implemented to promote procedural fairness of athletes which NADA and the Government of India could consider.

From the perspective of timeliness, it is clear that cases where 1000 days pass between the athlete being tested to a first-instance decision being reached are unacceptable.^123^ Indeed, the ISRM now prescribes timeliness as a guiding principle, whereby:

*In the interest of fair and effective sport justice, antidoping rule violations should be prosecuted in a timely manner. … Anti-Doping Organizations should be able to conclude Results Management (including the Hearing Process at first instance) within six (6) months from the notification [of the ADRV to the athlete].*^124^

In addition, the hearing process at first instance should take no longer than 2 months.^125^ Under the Code and corresponding International Standards, WADA is required to monitor NADOs efforts in implementing and complying with the applicable rules and regulations^126^ and there are mechanisms in place that allow WADA to hold NADOs accountable. For instance, under the ISRM, NADOs may face consequences if there are severe or systemic failures to comply with the mandatory timeliness requirements.^127^ This is consistent with the International Standard for Code Compliance by Signatories (ISCCS) which provides that WADA can hold NADOs accountable for non-compliance with the Code and ISRM.^128^ The ISCCS sets out several support mechanisms for NADOs to maintain compliance with obligations under the Code, including “providing advice and information, by developing resources, guidelines, training materials, and training programs, and by facilitating partnerships with other Anti-Doping Organizations where possible”.^129^ However, there are processes set out under the ISCCS for confirming non-compliance and imposing consequences on NADOs and other signatories to the Code.^130^

While there is still scope for a comprehensive empirical study on the timeliness of Indian anti-doping disputes, it is clear that numerous hearing procedures have exceeded this timeline.^131^ As such, NADA and the ADDP will need to adopt strict measures to ensure that the results management process, including hearings, are conducted within these strict time limits. This will require stricter scheduling of each stage of the results management process, including sample analysis and hearings. This may involve India’s domestic panels collaborating with anti-doping tribunals abroad to understand and emulate best practice standards in case management. The use of technology may improve efficiency in the hearing process, as it has in other jurisdictions where telephone and video hearings are common.^132^ In New Zealand, for example, telephone hearings have been driven by ‘logistical difficulties in arranging urgent hearings involving parties from around New Zealand and the considerable cost savings for all parties and, in particular, athletes’.^133^ If WADA were to push for compliance under the ISRM with respect to these time limits (and if there were consequences for systemic non-compliance), this may encourage first-instance panels and NADOs to ensure compliance.

Various jurisdictions have acknowledged the difficulties faced by athletes in finding affordable legal counsel and have established pro-bono counsel lists.^134^ Athletes can also apply for legal aid before the CAS.^135^ For first-instance hearings, the ISRM suggest that ‘the Results Management Authority and/or the relevant hearing panel should consider establishing a legal aid mechanism in order to ensure such access’.^136^ To date, no such legal aid mechanism exists in India. As a consequence, many athletes are unrepresented at first instance. In addition, the cost of requesting analytical laboratory reports, and engaging expert witnesses is prohibitively expensive for athletes in India.

Some commentators have argued that an overhaul of the entire sports dispute resolution process in India is required, noting that ‘it is the need of the hour to have an independent and separate institution for sports which is flexible and delivers quick and inexpensive resolution of sporting disputes’.^137^ This approach has been successful in several other jurisdictions.^138^ A National Sports Development Bill in 2013 proposed the creation of an Appellate Sports Tribunal. However, this Bill was not adopted by the Indian Parliament, which illustrates that there has been a lack of political will in the legislature to overhaul sports dispute resolution in India. Conversely, other countries have adopted necessary reforms to their sports dispute resolution system.^139^

Regardless of the inertia with respect to policy reform, as has been suggested previously,^140^ policymakers and scholars should conduct further empirical research to understand the extent that NADA and the ADDP have complied with the procedural guarantees and time limits prescribed under the NADA Rules and the Code.^141^ If empirical evidence shows that systemic issues exist in terms of timeliness and access to justice, this may be a catalyst for reform.

All three factors envisaged by Gray are represented in the procedural shortcomings of anti-doping disputes in India. First, WADA’s top-down approach places a heavy reliance on domestic bodies for implementation of procedure, as sanctions and accreditations are typically limited to testing, rather than procedural defects. Greater oversight and accountability to WADA may be necessary for jurisdictions who display systemic procedural difficulties in protecting the due process rights of athletes. The procedural shortcomings in anti-doping in India may also be a reflection of resource constraints of NADA and the domestic tribunals. Institutions in developing countries will invariably receive less funding from their national governments, and this remains one of the biggest challenges in uniformity in doping procedure. Greater funding may be required to implement capacity building and training programmes, case management policies and procedures, and other institutional reforms. Perhaps WADA can facilitate funding to jurisdictions who require further institutional investment and promote reform through its Regional Anti-Doping Organization (RADO) Program,^142^ in regions of particular concern (such as, for example, South Asia and Africa). Finally, systemic delays and access to justice issues may be a reflection of cultural nuances in India where civil and criminal litigation is notoriously slow and access to justice issues are widespread.^143^ Given the vision of harmonisation in anti-doping, WADA should work with countries such as India where such entrenched cultural legal processes exist to ensure that anti-doping procedures are an exception to these systemic domestic challenges, ensuring all athletes are afforded minimum protections when alleged of an anti-doping rule violation.

### Education and inadvertent doping

#### Education programmes

WADA’s Director of Education noted that ‘helping those bound by anti-doping rules to understand them; as well as, their rights and responsibilities is something WADA and our stakeholders must continue to commit to’.^144^ To this end, NADA has launched education programmes in India where various anti-doping workshops have been conducted in association with sports organisations and at colleges and universities.^145^ NADA has also translated anti-doping education material into 14 local languages so that athletes from across the country can understand it. Despite these initiatives from NADA, commentators remain critical of NADA’s education programmes given the lack of awareness among athletes on the risks of prohibited substances and their rights and responsibilities under the NADA Rules.^146^ Consistent with Gray’s (2019) framework, it is important to acknowledge that many NADOs ‘lack both human and financial resources, meaning that the priority is placed on the day-to-day administrative management rather than developing a wide-scale education programme’.^147^ However, if all athletes are to be held to such high standards as are prescribed under the Code, then all athletes have the right to be effectively educated about their rights and responsibilities under the Code.

India still consistently ranks amongst the worst countries with respect to ADRVs. While a proportion of these violations are likely to have been a result of intentional use, many may have resulted from misjudgements or lack of awareness of the risks of supplements and medicines used by athletes. Better education and awareness programmes are likely to reduce the incidence of doping in India.

Under the International Standard for Education, 2021 (ISE), NADOs are expected to ensure that athletes demonstrate competencies and skills “at each stage of their development”.^148^ Education programmes should be targeted towards athletes, support staff and coaches, sports administrators, as well as parents (in the context of minors) from grassroots to elite level. Studies suggest that “prevention programs are most effective when targeted at children and adolescents because attitudes and values are being formed during these stages of life.”^149^ Accordingly, consistent with the ISE, NADOs should identify target groups for their education programme,^150^ and such groups may include categories of young or adolescent athletes. For instance, WADA recommends that such target groups for anti-doping education may include emerging national-level athletes, younger athletes who are part of development teams or talent programmes, student-athletes in university sport and competitions, school children and even participants in recreational programs.^151^ With the establishment of the Khelo India School Games in 2018 and the Khelo India University Games in 2020,^152^ this may be an opportunity to promote anti-doping education to school and university students. Collaborating with education institutions (including the compulsory physical education classes in Indian schools),^153^ and grass roots sport institutes and academies may also enhance the reach of anti-doping education.^154^

The challenge of ensuring adequate anti-doping education is not unique to India—NADOs across the world should take measures to ensure that all athletes who are subject to dope testing understand the risks of doping and are aware of their rights and responsibilities under the Code. To ensure a comprehensive and effective education programme is in place, it is critical to promote collaboration with key stakeholders, especially national federations, who have regular contact with their athletes.^155^ While acknowledging that some federations have limited financial resources to implement education programmes (especially in developing countries),^156^ there is still scope for stronger collaboration with national federations in India to ensure that younger athletes, parents and support personnel are being educated at different stages of athlete development.^157^ Engaging with federations is not just a question of reaching more athletes (scale), it has also been argued that increased support from federations may result in “more education opportunities and increased engagement and enthusiasm” around engaging with the subject matter presented by NADOs.^158^ In India, however, there has not historically been a strong culture of education for athletes regarding the harmful effects of doping and the risks associated with doping regarding their sporting careers. This is especially true of athletes who have not competed at an international level. While NADA has initiated education programmes in India, there is still scope for improvement with respect to the promotion of anti-doping education to inculcate the value of dope free sports.

#### Inadvertent doping: contamination of supplements

The issue of inadvertent doping is often a symptom of both poor education and lack of institutional or regulatory reform. There have been several anti-doping cases before the CAS where athletes have claimed inadvertent doping due to use of medicines or dietary supplements wherein the athlete had no knowledge that the substance they consumed was banned, or it was contaminated.^159^

The use of dietary supplements by athletes is not uncommon, and there is a risk that such supplements may be contaminated with a prohibited substance, resulting in inadvertent doping.^160^ Due to several high-profile doping cases involving contaminated food supplements, the Indian Government explored ways in which athletes can consume nutritional supplements in a safe manner. In 2017, India’s Minister of Youth Affairs and Sports stated that:

*Tackling the causes of doping is a priority for the ministry. The import and sale of sub-standard and dope-laced nutritional supplements is a cause of worry as unsuspecting athletes get banned under the Anti-Doping Code because of use of these supplements*.^161^

The Ministry of Youth Affairs and Sports encouraged NADA and the Food Safety and Standards Authority of India (FSSAI) to cooperate in making nutritional supplements safe for all consumers, especially athletes. The FSSAI passed an order in 2017 clarifying that it is the responsibility of food business operators and manufacturers to ensure that health supplements do not contain any banned substances listed under the Code.^162^ Action may be taken against food business operators who include banned substances in their products, especially if such substances are not contained on the label of the product.^163^ The order encourages companies to seek clarifications from NADA and to ensure thorough testing of products before sale.^164^ The rationale of creating stricter standards for supplement manufacturers is that this would reduce the chances of contamination, thereby reducing the risk of accidental ingestion.

In addition, under the proposed National Anti-Doping Bill, 2021, NADA has the responsibility to:

*coordinate and collaborate with concerned authorities and stakeholders in matters relating to establishment of best practices in the marketing and distribution of nutritional supplements including information regarding their analytical composition and quality assurance.*^165^

It is also now responsible for “establishing standards for the manufacturing of nutritional supplements for sport in India.”^166^ Whether these orders (and the proposed additional responsibilities of NADA) have been implemented in practice remains to be seen. While in other jurisdictions, athletes have taken legal action against supplement companies for contamination resulting in an ADRV,^167^ no such cases have been reported in India. In any event, education and awareness of the potential risks of consuming supplements is paramount because under the Code, athletes may still face sanctions even if they can prove that the source of the prohibited substance is a contaminated supplement. Since arguments that high levels of inadvertent doping exist in India are to date mostly anecdotal, it is also recommended that evidence-based research is conducted to understand the proportion of athletes in India found to have committed an ADRV who claim to have doped inadvertently or accidentally. If the results of such empirical research are consistent with the several high-profile cases involving contamination of supplements and inadvertent doping, this may indeed point to more systemic issues requiring significant reform to domestic anti-doping policies, especially in the field of education.

### Legislative reform

While the NADA Rules are typically updated to align with the amended version of the Code,^168^ there has been some discussion about the need for legislative reform of the anti-doping framework in India. Recently, the National Anti-Doping Bill (2021), was tabled in Indian Parliament (Lok Sabha).^169^ The Bill aims to create a framework for institutional reform in anti-doping in India and proposes to streamline anti-doping authorities to encourage institutional and operational independence of anti-doping disputes.

If enacted, the Bill would establish a National Board for Anti-Doping in Sports (the Board) and a new National Anti-Doping Agency. The Bill proposes to give NADA additional powers, including the power to undertake inspections and search and seizure to determine any anti-doping rule violations.^170^ Hearings with respect to ADRVs are to be heard by the National Anti-Doping Disciplinary Panel^171^ and decisions from this panel may be appealed to the National Anti-Doping Appeal Panel.^172^ The Bill would make NADA and NDTL independent constitutional authorities, rather than under the control of the Ministry of Youth Affairs and Sports.^173^ This is consistent with WADA’s ISL which requires administrative and operational independence of laboratories to avoid potential conflicts of interest.^174^ It would also ensure compliance with the Code, which now requires NADOs to be operationally independent.^175^

It remains NADA’s responsibility to ensure that it conforms with the requirements under the Code and the international standards.^176^ Under the Bill, the Board is responsible for overseeing the activities of NADA, including with respect to “ensuring compliance with the anti-doping rules and standards laid down by [WADA].”^177^ In addition, the Board may call for information from the Disciplinary Panel and the Appeal Panel on its operations and issue directions “for the effective and timely discharge of their functions” insofar as such directions are limited to “procedural efficiency” without interfering with the decision-making process.^178^ Accordingly, the Bill provides the Board measures to hold NADA and domestic panels accountable for upholding principles of procedural fairness enshrined in the Code and the ISRM. This reform is significant as it provides athletes and legal counsel an avenue to report significant procedural issues caused by NADA, or the domestic anti-doping panels. However, it is important that the Board monitors (and enforces) requirements such as timeliness and access to legal representation and holds these bodies accountable for failures to meet any of the minimum procedural standards, and subsequently require procedural reform where severe or systemic procedural issues exist.

## Conclusion

While a key goal of WADA is to strive for harmonisation in the implementation of the anti-doping rules, the experience of anti-doping differs across countries. While there is a need to afford national governments and NADOs some autonomy in the implementation of the Code, the lack of harmonisation threatens the idea of creating a level playing field in anti-doping. This may in turn undermine the legitimacy of the entire framework.

The India case study highlights the need to conduct a deeper analysis with respect to how different countries implement the Code, and how the Code impacts athletes from different countries. Such empirical research may involve a content analysis of awards handed down by first-instance tribunals, and through primary research which engages with key stakeholders (such as athletes, arbitrators, and counsel) about their perceptions and experiences of the anti-doping system. This research will enable scholars and policy makers to ascertain whether the current framework has established the harmonised, level playing field that it intends to create. Further empirical research may also highlight, as some commentators have suggested,^179^ that the current framework disproportionately impacts athletes from developing countries.

While athletes are held to an extremely high standard under the Code—that of strict liability—the case studies discussed in this paper highlight the risks of anti-doping institutions not meeting the same high standards. As one panel noted:

*… just as the athletes who are subject to the anti-doping regime are expected to follow its rules and standards to the letter, so they are entitled to expect that those rules and standards will be strictly construed and followed by the anti-doping authorities themselves … Following the rules applicable to all stakeholders is the best method of ensuring the integrity of sport*.^180^

If anti-doping authorities do not meet the strict testing standards, education requirements, and procedural guarantees prescribed by the Code and International Standards, the consequences can be significant for athletes, as well as the perceptions of the legitimacy of the system.

In India, the effect of high-profile institutional failures undermines the confidence that athletes and other stakeholders have in the system at large. WADA’s suspension of the NDTL, allegations of glaring substantive errors by first-instance tribunals, highly publicised false positive and false negative tests, as well as legal challenges in High Courts on issues of procedural fairness and access to justice, have all added to the spout of controversies faced by the anti-doping institutions in India and globally. In addition, athletes rarely have recourse to institutions outside India, with only one of more than 1200 athletes having appealed to the CAS in more than a decade. While there has been some discussion by policymakers on how to improve the situation—by introducing a the National Anti-Doping Bill (2021) and through the regulation of supplements—there is plenty of scope for further reform to restore confidence in the system.

While improved education programmes are key across all jurisdictions, there is a need for improved institutional accountability across all facets of the process. WADA has taken measures to ensure compliance with strict international testing standards in India, and the NDTL and the Ministry need to ensure that such standards are improved moving forward. However, WADA, NADA and other stakeholders ought to strictly enforce the requirements under the Code with respect to procedural safeguards (such as timeliness and access to counsel), testing and education. While resources are limited in some jurisdictions, no athletes should be left to suffer due to institutional errors or inadequacies.

This paper has illustrated that some jurisdictions have not only fallen below the “best practice” standards set by NADOs and panels in some developed countries but have also failed to meet the minimum standards required under the Code (and corresponding International Standards). The Indian perspective, thus, emphasises that there is in fact a lack of harmonisation in implementing anti-doping rules and procedures across countries. Yet, if harmonisation is a desirable goal—as WADA emphasises—the Indian case study highlights that the global anti-doping framework is still far from reaching it.

## Data Availability

Not applicable.

## Appendix

See Fig. 1.

## Footnotes

1 Kambhampati and Star (2021).

2 WADA (2021a).

3 WADA (2021d).

4 Ibid.

5 Gatterer et al. (2020); Efverström and Bäckström (2017); Hanstad et al. (2010).

6 Batt (2011).

7 Dasgupta (2019); Star and Kelly (2021); Star and Kelly (2022).

8 Cf. Yang et al.’s (2021) paper where the authors seek to understand the implementation of anti-doping policies in China.

9 Very few cases involving Indian athletes have been appealed to the Court of Arbitration for Sport (CAS). To date, only 10 anti-doping cases have been appealed to the CAS involving 14 athletes: Star and Kelly (2021). This represents just over 1% of more than 1200 Indian athletes who have been found to have committed an anti-doping rule violation by the first-instance tribunal since its inception in 2009 (Star and Kelly 2022; NADA 2022). Star and Kelly (2021) further note that only one Indian athlete has appealed to the CAS for an anti-doping rule violation and that this data “may in itself be *prima face* evidence of access to justice issues in the anti-doping dispute resolution framework” (p. 110). While further empirical evidence is required to ascertain the cause of these accessibility constraints, the fact that the vast majority of cases are heard by first-instance tribunals in India underscores the importance of these bodies.

10 Kothari and Mehrotra (2019).

11 Vasavda (2020a).

12 Mohan (2020).

13 Sharma (2020). See, e.g., Dharam Raj Yadav v. NADA & Ors, Civil Writ Petition No. 8636 of 2020.

14 Food Safety and Standards Authority of India, Order dated 9 May 2017, “Use of Performance Enhancing Drugs (PED) in Health Supplements.” https://archive.fssai.gov.in/dam/jcr:1e78a364-c216-4715-8b33-b8852069c775/Order_Performance_Enhancing_Drug_08_06_2017.pdf. Accessed on 10 March 2022.

15 WADA Code (2021), p. 9.

16 WADA Code (2021), p. 16.

17 WADA Code (2021), Article 23.2.2. For instance, for the purposes of consistency and harmonisation, anti-doping organisations should base their decisions on (i) the same list of anti-doping rule violations; (ii) the same burdens of proof; and (iii) impose the same sanctions for the same anti-doping rule violations.

18 However, an athlete who is not an international-level athlete, or an athlete involved in an international event, may not necessarily have a right of appeal to the CAS. Rather, they will be permitted to appeal a first-instance decision to an appellate body in accordance with rules established by the National Anti-Doping Organization: WADA Code (2021), Article 13.

19 Hanstad et al. (2010).

20 Gray (2019).

21 Ibid.

22 Gray (2019), p. 255.

23 Ibid; Dimeo and Møller (2018); Efverström and Bäckström (2017); Park (2005).

24 Gray (2019), p. 255.

25 Ibid, p. 256; Houlihan (2013).

26 Gray (2019), p. 257.

27 Read et al. (2019), p. 234.

28 Pielke Jr. and Boye (2019), p. 297.

29 Ibid, p. 309.

30 NADA (2022): the total number of ADRVs is in accordance with the list published by NADA on 20 January 2022. See also, Table 1 and the Anti-Doping Testing Figures reported by WADA on an annual basis for further details on the total number of samples tested by NADA.

31 WADA (2017), p. 6.

32 WADA (2020a).

33 WADA (2019a)

34 The Hindu (4 April 2017).

35 Article 2.1 of the Code provides that “presence of a prohibited substance or its metabolites or markers in an athlete’s sample” constitutes an anti-doping rule violation. Note that under this provision, it is not necessary to show that the athlete had any intent, fault, or was negligent in consuming the prohibited substance, and as such this will result in an ADRV whether the prohibited substance was consumed intentionally or unintentionally.

36 Dayal (2018); Krishnan et al. (2022).

37 See, e.g., Dharam Raj Yadav v. NADA & Ors, Civil Writ Petition No. 8636 of 2020; Abhijeet Gurav v. NADA & Ors, Civil Writ Petition No. 616 of 2020; Kiran DevidasSanas v. NADA & Ors., Civil Writ Petition No. 1165 of 2020; Sachin Betkar v. NADA & Ors., Civil Writ Petition No. 1216 of 2020; Suresh Pai v. Union of India & Ors., Civil Writ Petition No. 1376 of 2020.

38 World Anti-Doping Code, International Standard for Laboratories, 2021, available at https://www.wada-ama.org/sites/default/files/resources/files/isl_2021.pdf.

39 International Standard for Testing, Article 1.1.1.

40 WADA (2019b). Discussed further in Sect. 2.2.2 below.

41 WADA (2020b), p. 1.

42 WADA (2021b).

43 Cyriac (2018).

44 See Table 1.

45 WADA (2018).

46 Article 4.6.4.

47 IRMS is an analytical technique required to be used by WADA-accredited laboratories before releasing of an AAF for the abuse of pseudoendogenous steroids (i.e., testosterone). See e.g., de la Torre et al. (2019).

48 Hussain (2019).

49 WADA (2019b).

50 Kothari and Mehrotra (2019); Mohan (2019).

51 Hussain (2019).

52 Vasavda (2020a).

53 Kothari and Mehrotra (2019).

54 Inderjeet Singh v. NADA, Anti-Doping Appellate Panel, Case No. 08, ADAP 2018, para 8.

55 Vijay Singh v. National Anti-Doping Agency (NADA) & Anr., Delhi High Court, W.P.(C) 3766/ 2021.

56 Singhania and Dhingra (2021).

57 Vijay Singh v. National Anti-Doping Agency (NADA) & Anr., W.P.(C) 3766/2021, Delhi High Court, order dated 22 March 2021 (Justice Prathiba M. Singh), http://delhihighcourt.nic.in/dhcqrydisp_o.asp?pn=48509&yr=2021.

58 Das (2021).

59 Vijay Singh v. National Anti-Doping Agency, Anti-Doping Appeal Panel, Case number- 04.ADAP.2020.

60 Ibid.

61 The Economic Times (23 August 2019).

62 Mohan (2019).

63 WADA (2021c).

64 NADA Rules (2021), Article 8.1.

65 NADA Rules (2021), Articles 13.2.1 and 13.2.2.

66 NADA Rules (2021), Article 8.5.

67 Star and Kelly (2021).

68 Rajaraman (2020). See NADA vs Himanshu Kumar Chang (Case No. 74.ADDP.04.2019), award of 6 January 2020 and NADA vs Vishal Solanki (Case No. ADDP.2020), award of 27 May 2020. It is interesting to note that while the ADAP upheld the original ADDP decision, the award states that the ADDP order should be “modified” by striking out the erroneous section of the judgment that was incorrectly copied from the facts of the Himanshu Kumar Chang case (Vishal Solanki vs National Anti-Doping Agency (NADA), Appeal No. 04.ADAP.2020, Award dated 15 July 2021).

69 Mohan (2020). See, e.g., NADA vs Mukul Sharma (Case No. 145.ADDP.01.2019), award of 17 December 2019; NADA vs Himanshu Kumar Chang (Case No. 74.ADDP.04.2019), award of 6 January 2020.

70 Mohan (2020).

71 Ibid.

72 WADA Code (2021), Article 8; ISRM (2021), Article 8.

73 Star and Kelly (2021), p. 96.

74 Kambhampati and Star (2021).

75 WADA Code (2021), Article 13.2.2; ISRM (2021), Article 8.8(d).

76 Star and Kelly (2021), p. 107; Dimeo and Møller (2018), p. 193; Weston (2009), p. 49; Kambhampati and Star (2021), p. 234.

77 Dasgupta (2019); See also, Star and Kelly (2022).

78 Star (2022).

79 Star and Kelly (2021).

80 WADA Code (2021), Article 8.

81 Kambhampati and Star (2021), p. 234.

82 Amar Muralidharan v. Indian National Anti-Doping Agency of India, Indian National Dope Testing Laboratory, Ministry of Youth Affairs and Sports (CAS 2014/A/3639), award of 8 April 2015; World Anti-Doping Agency v. Amit and National Anti-Doping Agency of India (CAS 2014/A/3869), award of 23 November 2015, para 63; World Anti-Doping Agency v. Nirupama Devi Laishram and National Anti-Doping Agency of India (CAS 2012/A/2979), award of 8 November 2013, paras 119–120.

83 NADA v. Anil Kumar (Case No. 43.ADDP.01.2012), award of 17 December 2012.

84 Ibid.

85 Woolf (2020).

86 Dayal (2018).

87 WADA Code (2021), Article 2.1.1.

88 Dayal (2019).

89 The Economic Times (6 July 2011).

90 Gatterer et al. (2020), p. 230; Backhouse et al. (2014).

91 Dayal (2018).

92 Ibid.

93 Krishnan et al. (2022).

94 Ibid.

95 Ibid.

96 Kambhampati and Star (2021).

97 Dharam Raj Yadav v. NADA & Ors, Civil Writ Petition No. 8636 of 2020; Abhijeet Gurav v. NADA & Ors, Civil Writ Petition No. 616 of 2020; Kiran DevidasSanas v. NADA & Ors., Civil Writ Petition No. 1165 of 2020; Sachin Betkar v. NADA & Ors., Civil Writ Petition No. 1216 of 2020; Suresh Pai v. Union of India & Ors., Civil Writ Petition No. 1376 of 2020. It should be noted that injunctions have been ordered by the High Court, where required, and full substantive judgments are still awaited. For instance, in Abhijeet Gurav v. NADA & Ors, Civil Writ Petition No. 616 of 2020, the High Court has heard appearances from counsel 24 times since the initial hearing on 17 January 2020 until 16 February 2022. A final determination is still pending.

98 Dharam Raj Yadav v. NADA & Ors, Civil Writ Petition No. 8636 of 2020.

99 Ibid.

100 Ibid.

101 Ibid, para 18.

102 See NADA Rules (2021), Comment to Article 7.2.

103 Moston and Engelberg (2019), p. 261.

104 International Association of Athletics Federation (IAAF) v. Eddy Hellebuyck, Arbitration CAS 2005/A/831, award of 5 May 2006, para 21.

105 WADA Code (2021), Comment to Article 3.2.2.

106 Pielke Jr. and Boye (2019).

107 Amar Muralidharan v. Indian National Anti-Doping Agency (NADA), Indian National Dope Testing Laboratory, Ministry of Youth Affairs and Sports (CAS 2014/A/3639), award of 8 April 2015, para 88.

108 Ibid, para 18.

109 Ibid, para 88.

110 Ibid, para 91.

111 NADA (2021).

112 Gray (2019); Houlihan (2013).

113 WADA (2010); Doping Control Centre, Universiti Sains Malaysia v. World Anti-Doping Agency (WADA), Arbitration CAS 2010/A/2162, award of 15 June 2011; Pielke Jr. and Boye (2019).

114 See, International Standard for Laboratories, Article 4.6.4.1 (Suspension of Accreditation and Analytical Testing Restriction); Article 4.6.4.2 (Noncompliances with the ISL); Article 4.6.4.3 (Revocation of Accreditation).

115 It is estimated that ‘NDTL [charges] $250. When sent abroad, the price per sample goes up to $350, excluding the transportation costs’: Vasavda (2020b).

116 PTI (23 August 2019); Vasavda (2020b).

117 Gray (2019); Houlihan (2013).

118 Müller (2017), p. 186.

119 WADA (2016a).

120 Similarly, the Ministry should increase its investment in capacity building and case management with respect to the ADDP and ADAP.

121 Star and Kelly (2021).

122 Amar Muralidharan v. Indian National Anti-Doping Agency of India, Indian National Dope Testing Laboratory, Ministry of Youth Affairs and Sports (CAS 2014/A/3639), award of 8 April 2015.

123 NADA v. Anil Kumar (Case No. 43.ADDP.01.2012), award of 17 December 2012.

124 ISRM (2021), Article 4.2.

125 ISRM (2021), Article 8.8.(c).

126 See WADA Code (2021), Article 20.7.2; International Standard for Code Compliance by Signatories (ISCCS), Article 8.

127 See ISRM, Comment to Article 4.2.

128 The strict minimum procedural standards under the WADA Code and ISRM are identified as a high priority to anti-doping procedures as set out in ISCCS, Annex A, A.1.2(l).

129 ISCCS, Article 7.1.

130 ISCCS, Article 10. See also WADA Code (2021), Article 24.1. The possible consequences that may be imposed on NADOs for violating the procedural fairness provisions (and others) enshrined in the WADA Code and International Standards are set out in Article 24.1.12 of the WADA Code (2021).

131 See, e.g., Amar Muralidharan v. Indian National Anti-Doping Agency of India, Indian National Dope Testing Laboratory, Ministry of Youth Affairs and Sports (CAS 2014/A/3639), award of 8 April 2015; NADA v. Anil Kumar (Case No. 43.ADDP.01.2012), award of 17 December 2012. See, also, Star and Kelly (2022).

132 Note that while video hearings before the ADDP have been conducted during the COVID-19 pandemic in India, these were not commonplace earlier.

133 David (2016), p. 141.

134 For instance, National Sports Tribunal (Australia), Legal Assistance Panel, https://www.nationalsportstribunal.gov.au/dispute-resolution-services#nst-legal-assistance-panel-nstlap; Sports Resolutions UK, http://www.sportresolutions.co.uk/services/pro-bono-legal-advice; Sports Tribunal of New Zealand, http://www.sportstribunal.org.nz/rules-and-procedures/legal/.

135 CAS (2020).

136 ISRM (2021), Comment to Article 8.8(b).

137 Singhania and Kothari (2021).

138 Including in the UK (Sport Resolutions provide independent dispute resolution in sport, including in anti-doping matters under the National Anti-Doping Panel); in New Zealand (the Sports Tribunal of New Zealand); in Canada (the Sport Dispute Resolution Centre); in Australia (National Sports Tribunal).

139 For instance, Star and Kelly (2021), noting that ‘… New Zealand’s Sports Anti-Doping Rules have been amended every year over the past decade,’ and that ‘Australia’s National Sports Tribunal in March 2020 has drawn on the policies and experiences of dispute resolution frameworks in other developed countries such as Canada, New Zealand and the United Kingdom.’

140 Star and Kelly (2021).

141 See, e.g., Star and Kelly (2022).

142 According to WADA, the objectives of the RADO program are (i) sustainability and accountability; (ii) capacity building; (iii) compliance and program development; (iv) relations and engagement. See https://www.wada-ama.org/en/rado-program.

143 Krishnan et al. (2014), Rehn et al. (2011).

144 WADA (2019c).

145 Press Information Bureau (2016).

146 Dayal (2018).

147 Cléret (2011), p. 276.

148 ISE, Article 5.4,

149 Gatterer et al. (2020), p. 230. See also, Pöppel (2021).

150 ISE, Article 4.2.2.

151 WADA (2020c).

152 Ministry of Youth Affairs and Sports, Government of India, Khelo India University Games. https://universitygames.kheloindia.gov.in/. Accessed 8 March 2022.

153 Ministry of Education (2019) Several steps have been taken by the Government to impart health and physical education to students across the country : HRD Minister. 5 December 2019. https://pib.gov.in/PressReleasePage.aspx?PRID=1595137#:~:text=CBSE%20has%20made%20Health%20and,classes%20I%2DXII%20each%20day. Accessed 8 March 2022.

154 For example, Pöppel (2021) notes that “there is an effort to integrate anti-doping education into the German curricula in cooperation with the national anti-doping agency”, citing Klüttermann (2019).

155 Gray (2019).

156 Winand (2015); Gatterer et al. (2020).

157 See e.g., WADA (2020c).

158 Gatterer et al. (2020), p. 235.

159 See e.g., Flavia Oliveira v. United States Anti-Doping Agency (USADA), award of 6 December 2010, Arbitration CAS 2010/A/2107; Arijan Ademi v. Union of European Football Associations, CAS 2016/A/4676.

160 McArdle (2015). See also, Gatterer et al. (2020), Martinez-Sanz et al. (2017).

161 The Times of India (30 June 2017).

162 Food Safety and Standards Authority of India, Order dated 9 May 2017, “Use of Performance Enhancing Drugs (PED) in Health Supplements”, available at https://archive.fssai.gov.in/dam/jcr:1e78a364-c216-4715-8b33-b8852069c775/Order_Performance_Enhancing_Drug_08_06_2017.pdf. A list of banned substances are annexed to the FSSAI Order.

163 Ibid.

164 Ibid.

165 The National Anti-Doping Bill (2021), Article 6(3)(j).

166 Ibid, Article 6(3)(j).

167 For example, US athletes Jessica Hardy and William Frullani, see Abrahamson (2009); Krolak (2015).

168 See NADA Rules (2009), NADA Rules (2015), NADA Rules (2021).

169 Hussain (2022).

170 The National Anti-Doping Bill (2021), Article 19(1)(a). In exercising the power of entry, search and seizure, anti-doping authorities must comply with the provisions of the Code of Criminal Procedure, 1973.

171 Ibid, Article 21.

172 Ibid, Article 23.

173 Hussain (2020b).

174 ISL (2021), Article 4.4.2.4.

175 WADA Code (2021), Article 20.5.1.

176 The National Anti-Doping Bill (2021), Article 16(3)(e)

177 Ibid, Article 10(3)(a)

178 Ibid, Article 10(5).

179 Star and Kelly (2021); Dasgupta (2019).

180 USADA v. Latasha Jenkins (2008), AAA No. 301900019907 (American Arbitration Association, award dated 25 January 2008), para 161.
